# Ablating hedgehog signaling in tenocytes during development impairs biomechanics and matrix organization of the adult murine patellar tendon enthesis

**DOI:** 10.1002/jor.22899

**Published:** 2015-04-14

**Authors:** Andrew P. Breidenbach, Lindsey Aschbacher‐Smith, Yinhui Lu, Nathaniel A. Dyment, Chia‐Feng Liu, Han Liu, Chris Wylie, Marepalli Rao, Jason T. Shearn, David W. Rowe, Karl E. Kadler, Rulang Jiang, David L. Butler

**Affiliations:** ^1^ Department of BiomedicalBiomedical Engineering ProgramChemical and Environmental EngineeringUniversity of CincinnatiCincinnatiOhio; ^2^ Division of Developmental BiologyCincinnati Children's Hospital Medical CenterCincinnatiOhio; ^3^ Wellcome Trust Centre for Cell‐Matrix ResearchFaculty of Life SciencesUniversity of ManchesterManchesterUK; ^4^ Department of Reconstructive SciencesSchool of Dental MedicineUniversity of Connecticut Health CenterFarmingtonConnecticut; ^5^ Department of Cellular & Molecular MedicineCleveland Clinic Lerner Research InstituteClevelandOhio

**Keywords:** enthesis, tendon mechanics, fibrocartilage, mineralization, hedgehog, serial block face‐scanning electron microscopy

## Abstract

Restoring the native structure of the tendon enthesis, where collagen fibers of the midsubstance are integrated within a fibrocartilaginous structure, is problematic following injury. As current surgical methods fail to restore this region adequately, engineers, biologists, and clinicians are working to understand how this structure forms as a prerequisite to improving repair outcomes. We recently reported on the role of Indian hedgehog (Ihh), a novel enthesis marker, in regulating early postnatal enthesis formation. Here, we investigate how inactivating the Hh pathway in tendon cells affects adult (12‐week) murine patellar tendon (PT) enthesis mechanics, fibrocartilage morphology, and collagen fiber organization. We show that ablating Hh signaling resulted in greater than 100% increased failure insertion strain (0.10 v. 0.05 mm/mm, *p*<0.01) as well as sub‐failure biomechanical deficiencies. Although collagen fiber orientation appears overtly normal in the midsubstance, ablating Hh signaling reduces mineralized fibrocartilage by 32%, leading to less collagen embedded within mineralized tissue. Ablating Hh signaling also caused collagen fibers to coalesce at the insertion, which may explain in part the increased strains. These results indicate that Ihh signaling plays a critical role in the mineralization process of fibrocartilaginous entheses and may be a novel therapeutic to promote tendon‐to‐bone healing. © 2015 The Authors. *Journal of Orthopaedic Research* published by Wiley Periodicals, Inc. on behalf of the Orthopaedic Research Society. J Orthop Res 33:1142–1151, 2015.

Tendon and ligaments attach to bone to effect joint motion and stability in response to strong and impulsive muscle forces. Injuries to these tissues often occur at their insertion into bone (i.e., enthesis), and integration of the soft graft tissue with the bone following surgical reconstruction, remains a challenge. Furthermore, these current methods do not reduce the risk of re‐injury.^1^ Tissue engineering using mesenchymal progenitor cells has shown promise in repairing the tendon midsubstance.^2^ However, these techniques have failed to regenerate the native structure and organization of the tendon enthesis.^3^, ^4^

Type I collagen‐containing fibrils are the most abundant and well‐organized protein polymer in tendon. These fibrils are approximately parallel to the long axis of the tendon midsubstance and are organized in a hierarchical system of fibers (also referred to as “fibril bundles”) and fascicles (closely packed fibers).^5^ The enthesis is the transitional structure where collagen fibrils, fibers, and fascicles of the midsubstance transition through a region of fibrocartilage before inserting into the bone.^6^ The zonal insertion also contains both unmineralized and mineralized fibrocartilage, characterized by a tidemark delineating these regions. These two regions vary in extracellular matrix composition and organization, including mineral content and collagen types.^7^, ^8^ Furthermore, the orientation of collagen fibers through the enthesis is critical for efficient load transmission.^9^ Following avulsion of tendon from the bone, natural healing, traditional surgical repair, and current tissue engineering strategies result in disorganized scar tissue that does not restore this integrated network of aligned collagen fibers embedded in mineralized fibrocartilage.^3^, ^4^, ^10^, ^11^ Such disorganized scar tissue likely explains the increased insertion strains that persist into later stages of healing.^12^ Understanding the normal development and maturation of these structures may lead to novel insights into how to address the limitations of current repair techniques. However, the process by which the fibrocartilage structure develops and matures has been less well documented.

We recently reported on a novel role that hedgehog (Hh) signaling plays in the development of the patellar tendon insertion site.^13^, ^14^ Initial experiments showed that Hh signaling was co‐localized with Scx‐expressing cells specifically at the murine distal patellar tendon insertion during early postnatal development.^13^ Through in vitro and in vivo gain‐of‐function experiments, we showed that constitutive activation of Hh signaling in Scx‐expressing cells leads to ectopic expression of insertion site extracellular matrix proteins within the tendon midsubstance.^14^ Additionally, loss‐of‐function experiments were conducted by ablating Hh signaling in Scx‐expressing cells. Results illustrated reduced expression of chondrogenic and mineralization markers in the enthesis as early as 2 weeks of age. These reductions in proteoglycan content lead to apparent dysfunction in tidemark formation which persisted until 12 weeks of age. Hh ablation additionally generated full‐length biomechanical deficiencies in the adult tendon. However, it is unclear if abnormalities of insertion site formation caused increased strains locally at the insertion site. Therefore the objective of this study was to identify how ablating Hh signaling in Scx‐expressing cells affects regional biomechanical properties, fibrocartilage maturation and collagen fiber organization in the adult murine patellar tendon. Based on our previous findings, we hypothesized that ablating Hh signaling in the patellar tendon during development would result in mature tendons exhibiting increased insertion strain and disrupted fiber organization within the mineralized fibrocartilage.

## MATERIALS AND METHODS

### Animal Breeding

All husbandry procedures were approved by the Institutional Animal Care and Use Committee at CCHMC. Tenocyte‐specific knockout animals (Smo tKO) and wildtype littermates (controls) were used in this study. We targeted the cell surface receptor smoothened (Smo), which mediates activation of the Hh signaling pathway.^15^ Tendon cells express the transcription factor Scleraxis (Scx) throughout tendon development and maturation.^16^, ^17^, ^18^ We used a Cre‐Lox breeding scheme, crossing Scx‐Cre with Smo‐floxed mice, to generate ablation of Hh signaling in Scx‐expressing tendon cells as previously reported.^14^ Offspring were genotyped via PCR using primers for Cre, Smo wild‐type allele, and Smo floxed allele as previously described.^14^ Smo tKO mutants and controls were euthanized by CO_2_ and cervical dislocation for analysis.

### Experimental Design

The patellar tendons (PT) of 12‐week old male Smo tKO mice (*n* = 12) were compared to wild‐type controls (*n* = 12) for biomechanics, histological structure, and collagen fiber organization using second harmonic generation (SHG) microscopy and serial block face‐section scanning electron microscopy (SBF‐SEM). SHG microscopy was used to evaluate gross tissue‐level collagen fiber organization, whereas SBF‐SEM provided higher resolution to evaluate fiber‐to‐fiber interactions. One hindlimb of each mouse was assigned for biomechanical testing (*n* = 12), and a subset of contralateral limbs was randomly selected for histological/SHG (*n* = 3) or SBF‐SEM (*n* = 1) analysis.

### Biomechanics

**Figure 1:**
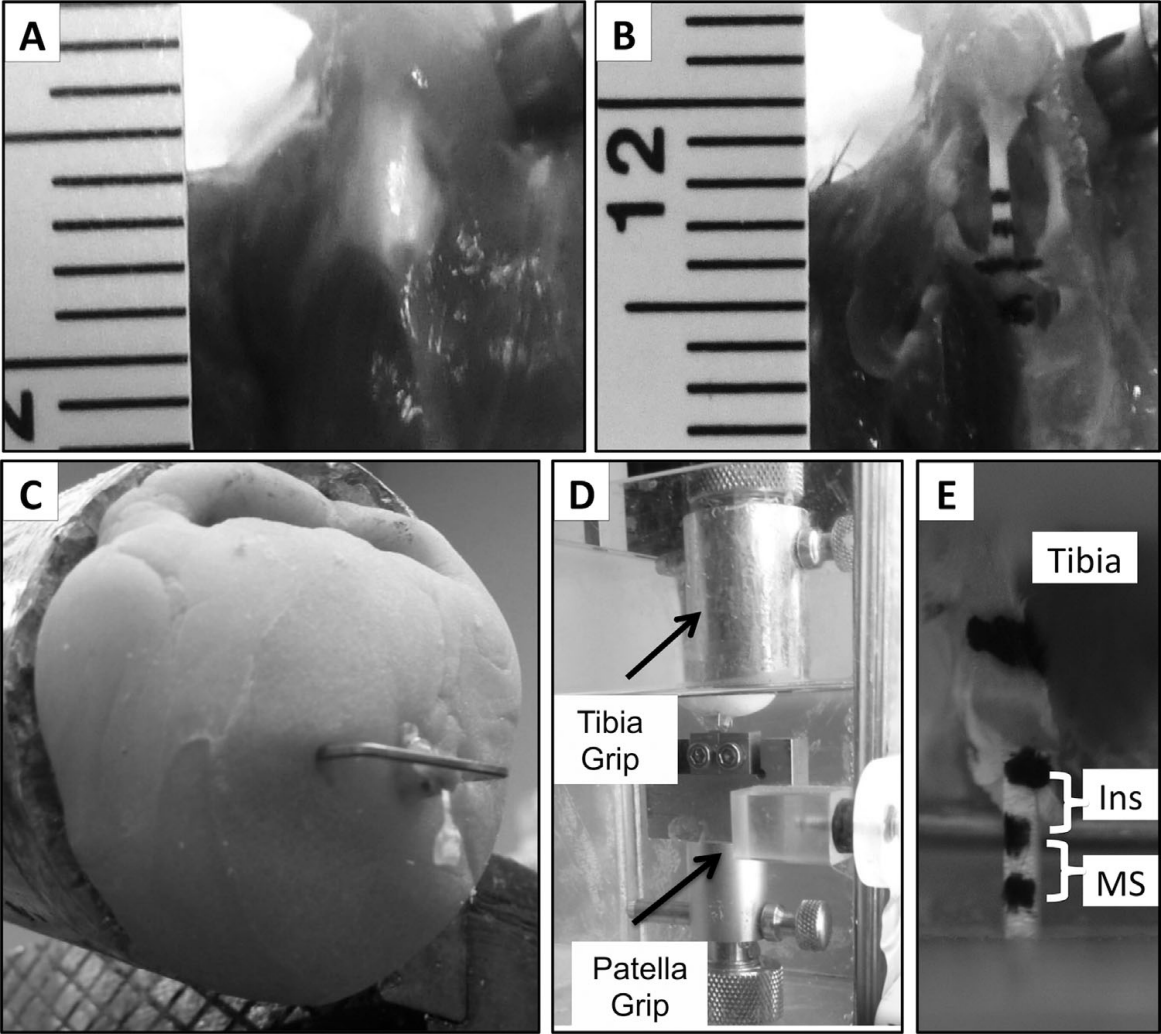
Biomechanical testing procedure. Hind limbs were dissected to expose patellar tendons (A) and the central portion of the patellar tendon (PT) was harvested by removing medial and lateral struts (B). Verhoeff's stain was applied to the tibia (rigid mark to track total tendon displacement) and to anterior surface of PT to delineate insertion and midsubstance regions for optical tracking (B). Patella‐PT‐tibia units were further dissected and mounted using bone cement in a custom grip and stabilized with a staple over the tibial plateau (C). Samples were then loaded into a materials testing system (D), and images were captured for optical tracking of total tendon displacement (tibial mark) and regional insertion site (Ins) and midsubstance (MS) deformations (E). Methods courtesy of Dr. Lou Soslowksy (UPenn; see Dourte et al., J Biomech Eng, 2012^19^).

Biomechanical testing was performed as previously described.^10^, ^12^, ^19^ Briefly, central width (48.7 ± 2.7% of full width) patella‐PT‐tibia units were dissected from each animal (Fig. 1A). Suture soaked with Verhoeff's stain was used to create transverse optical marks on the tibia and in three locations along the anterior surface of the tendon (Fig. 1B). These marks allow for tracking of whole tendon and regional (midsubstance and insertion) deformations, respectively. Tendons were inserted into custom grips in a 37°C 1X phosphate buffered saline (PBS) bath and preloaded in tension to 20 mN in a material testing machine (Test Resources; Shakopee, MN; Fig. 1C and D). Calibrated digital images were taken in coronal and sagittal planes to determine tendon width and thickness, respectively, from which cross‐sectional area was computed (Fig. 1E). Specimens were preconditioned (25 cycles, 0–1% strain, 0.003 mm/s) and then failed in tension at a constant displacement rate (0.003 mm/s). High resolution (≤6 µm/pixel) images were captured during failure testing at 5 s intervals using a Tamron macro lens (model SPAF90; Commack, NY) on a Nikon DSLR camera (model D5100; Melville, NY). Gray‐scale images were imported into FIJI (v. 1.48p) and thresholded to binary images, and the MTrack2 plug‐in was used to extract deformation data from binary images. Optical displacement, optical regional strain and load data were interpolated using 3rd degree polynomial basis spline (B‐spline) techniques to model the failure curves using the R statistical package.^20^, ^21^ Briefly, regional strains were modeled for each specimen using linear combinations of piecewise cubic polynomials according to the formula:

(1) $$f(x)=\Sigma_{i=1}^{13}\beta_{i}*\alpha_{i}(x)$$

for 0 ≤ *x* ≤ 100, where *x* is the percent of failure load/stress, α*_i_*(*x*) are cubic polynomials (as defined by the B‐spline model in R), and *β_i_* are coefficients on overlapping intervals (illustrated in Fig. S1). Each *β_i_* coefficient varied from specimen to specimen to describe sub‐failure mechanical behavior (e.g., regional strain v. load). Coefficients were then averaged across specimens to generate a model function that captures the behavior of a given treatment group. Derivatives of B‐splines with respect to displacement and strain were used to model instantaneous stiffness and modulus of structural and material failure curves, respectively. B‐spline coefficients were statistically evaluated to identify where sub‐failure deviations existed across treatments (see Section Collagen Fiber Organization Using SBF‐SEM).

### Histology

#### Sample Preparation and Imaging

After removing skin, the hindlimb was fixed in 4% paraformaldehyde (Electron Microscopy Sciences; Hatfield, PA) in 1X PBS overnight at 4°C. Samples were next washed three times in 1X PBS for 15 min, preserved in 30% sucrose in 1X PBS overnight at 4°C, and then embedded in OCT media (Andwin Scientific; Addison, IL) and frozen at −80°C. Sagittal cryosections (20‐µm) were cut on a Reichert‐Jung Cryocut 1800 (Depew, NY) using a cryofilm process (Type 2C; Hiroshima, Japan) as previously described.^22^ Hydrated sections were mounted in propidium iodide (PI, 1:1000; Sigma‐Aldrich) in 1X PBS immediately prior to multi‐photon (MP) imaging. Sections were imaged in three regions (patella, midsubstance, and tibial insertion) with a Nikon A1R upright MP laser scanning microscope (Melville, NY) and a TiSapphire laser to evaluate collagen fiber organization using second harmonic generation (SHG).^23^ Images were taken using a 25X water‐immersion objective in galvanometric mode for SHG signal (400–450 nm) and PI counterstain (595–645 nm) while maintaining consistent laser settings across all samples. After MP imaging, sections were washed briefly in ddH_2_O, stained with Toluidine Blue O (TBO, 1:1000; Sigma‐Aldrich; St. Louis, MO) for 45 s, rinsed in ddH_2_O, and then mounted in 50% glycerol in ddH_2_O. Colorimetric images were taken at 10× (Zeiss Apotome; Thornwood, NY) in wide‐field mode and stitched together using FIJI's grid stitching plug‐in.^24^

#### Image Quantification

Three (*n* = 3) sagittal sections were taken from the central region of each specimen (9 total sections for each genotype) for quantitative analysis. Calibrated images were imported into FIJI using the Bio‐Formats plug‐in (v. 4.4.9). TBO images were used to measure the size of the mineralized FC and the angle of the tidemark with respect to the long tendon axis. Mineralized FC size was estimated by measuring the cross‐sectional area in each section, and the tidemark angle was measured between the anterior‐posterior tangent line along the interface of the unmineralized and mineralized FC (the tidemark) and the longitudinal axis of the tendon midsubstance. Collagen fiber orientation of the mineralized and unmineralized insertion was calculated using the FIJI plug‐in OrientationJ, and each sample was normalized to the average orientation of the midsubstance to correct for any deviations in sample alignment in the microscope's field of view.

### Collagen Fiber Organization Using SBF‐SEM

#### Sample Preparation and Imaging

Hind limb skin was removed, and each limb was placed in fixative (2.5% glutaraldehyde, 0.1M cacodylic acid, 0.2M phosphate buffer; Electron Microscopy Sciences) for 4 h at 4°C. Samples were then transferred to fresh fixative containing 0.25M EDTA and changed every 2 days for one week to decalcify the mineralized tissue. Samples were then washed three times in phosphate buffer for 10 min each. The central portion of the tendon was dissected by removing the medial and lateral edges under a dissecting microscope, and regions corresponding to the midsubstance and insertion (as defined by the dye lines used for biomechanical analysis) were isolated and further processed for serial block face‐scanning electron microscopy (SBF‐SEM) as previously described.^25^

#### Three Dimensional Reconstructions

IMOD software (v. 4.8.16) was used to process SBF‐SEM datasets as previously reported.^26^, ^27^ Briefly, collagen fibers were outlined in successive z‐plane frames and reconstructed to illustrate overall bundle shape over ∼70‐µm along the tissue axis.

### Statistics

Student's *t* tests were used to evaluate the effects of genotype on all biomechanical (linear stiffness/modulus, failure properties, sub‐failure regional strains, regional strain rates, β coefficients) and histological (fiber orientation, mineralized FC size, tidemark angle) quantitative measures (*p* < 0.05). β coefficients for optical failure strains were heteroscedastic and were thus compared with Welch's *t* test (*p* < 0.05). All values reported are mean ± SEM.

## RESULTS

### Tissue Dimensions and Biomechanical Analysis

**Figure 2:**
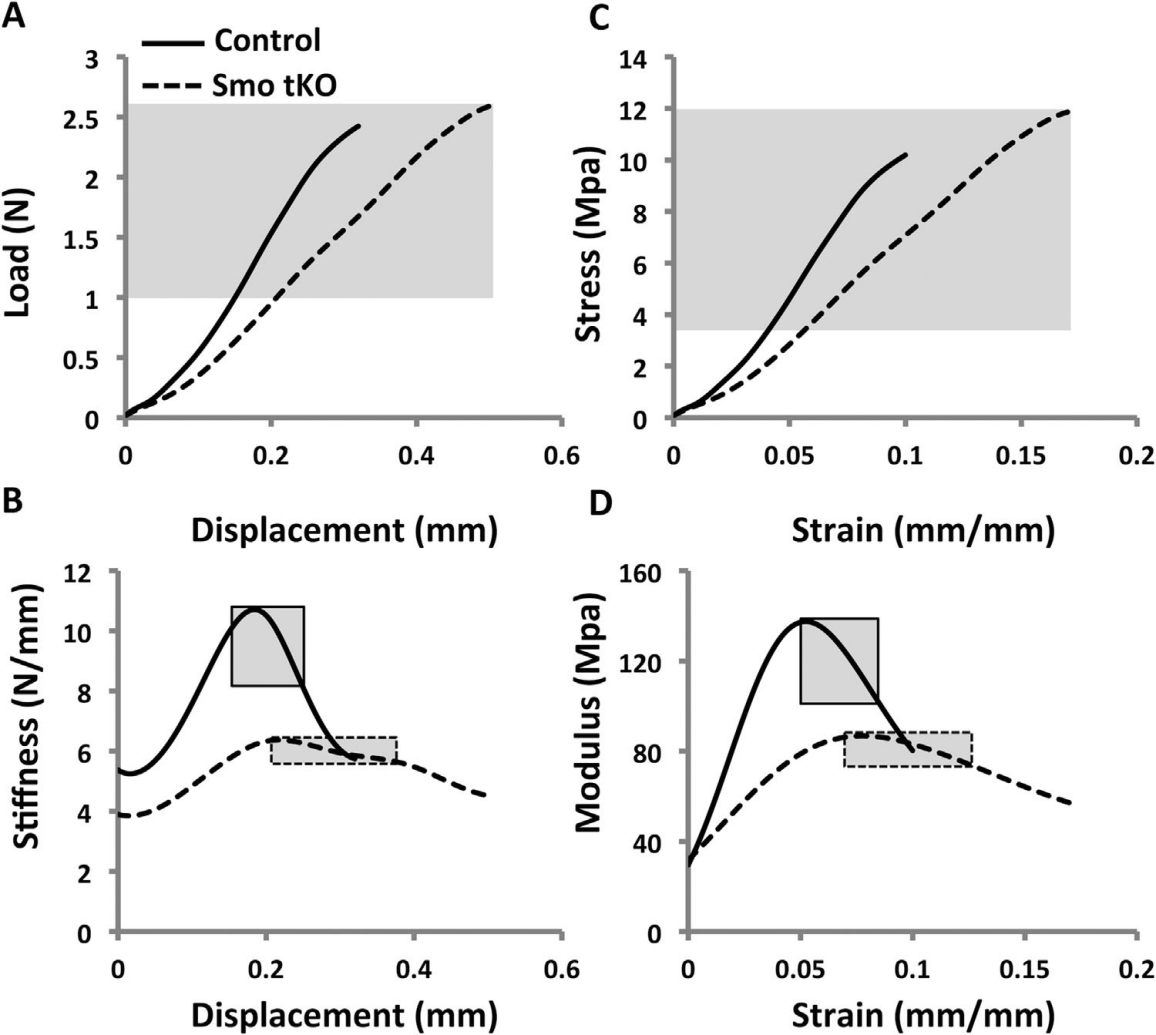
Ablation of Hh signaling during development causes abnormal adult patellar tendon mechanics. B‐spline models of structural data (A), instantaneous stiffness (B), material data (C) and instantaneous modulus (D). Compared to controls, Smo tKO tendons exhibited increased tissue displacement above 40% of failure loads (gray shaded region in A), resulting in 36% increased tissue displacement at failure (A). Smo tKO tendons showed ∼34% reduced linear stiffness in the 40–80% of failure load range (B). Smo tKO tendons also showed 50% greater failure strain than controls beginning at 30% of ultimate stress (C), resulting in 38% lower linear modulus in the 40‐80% range of ultimate stress (D).

Ablating Hh signaling affected biomechanical properties of the whole patellar tendon as well as locally at the insertion site. Although Smo tKO mice (29.8 ± 1.1 g) were approximately 13.6% smaller than control mice (34.5 ± 1.0 g), tendon length (*p* = 0.18), full width (*p* = 0.28), thickness (*p* = 0.43), and dissected width (*p* = 0.46) did not significantly differ between Smo tKO and controls (Table 1). Despite dimensional similarities in full‐length tendons, Smo tKO tendons showed a 34% reduction in linear stiffness (7.6 ± 0.7 N/mm v. 11.5 ± 1.1 N/mm, *p* < 0.01) and a 36% increase in failure displacement (0.50 ± 0.06 mm v. 0.32 ± 0.03 mm, *p* < 0.05) compared to controls, respectively (Table 1; Fig. 2A and B). Smo tKO tendons showed similar differences for material properties, including a 38% lower linear modulus (103.3 ± 8.3 MPa v. 167.5 ± 19.9 MPa, *p* < 0.05) and 70% greater strain at failure (0.17 ± 0.02 mm/mm v. 0.10 ± 0.01 mm/mm, *p* < 0.05) compared to controls, respectively (Table 1; Fig. 2C and D). Maximum failure force (2.64 ± 0.27 N v. 2.46 ± 0.19 N, *p* = 0.59) and ultimate stress (12.08 ± 1.10 MPa v. 11.04 ± 0.75 MPa, *p* = 0.44) did not differ between mutants and controls, respectively (Table 1; Fig. 2A and C).

**Table 1:** Patellar Tendon Dimensions and Biomechanical Properties

	Control	Mutant	*p* value
Weight^a^ (g)	34.5 ± 1.0	29.8 ± 1.1	<0.01
Full‐length measurements			
Linear stiffness^a^ (N/mm)	11.5 ± 1.1	7.6 ± 0.7	<0.02
Max. failure force (N)	2.4 ± 0.2	2.6 ± 0.3	0.61
Max. displacement^a^ (mm)	0.32 ± 0.03	0.50 ± 0.06	0.02
Elastic modulus^a^ (MPa)	167.5 ± 19.9	103.3 ± 8.3	0.01
Max. stress (Mpa)	10.9 ± 0.7	11.9 ± 1.1	0.46
Max. strain^a^ (mm/mm)	0.10 ± 0.01	0.17 ± 0.02	0.02
Length (mm)	3.16 ± 0.09	2.99 ± 0.09	0.18
Full width (mm)	1.15 ± 0.06	1.07 ± 0.03	0.28
Dissected width (mm)	0.52 ± 0.02	0.54 ± 0.02	0.46
Thickness (mm)	0.44 ± 0.03	0.41 ± 0.02	0.42
Regional measurements			
Midsubstance			
Max. strain (mm/mm)	0.06 ± 0.01	0.05 ± 0.01	0.53
Region length total length (%)	24.9 ± 0.9	25.5 ± 1.2	0.71
Insertion			
Max. strain^a^ (mm/mm)	0.05 ± 0.01	0.10 ± 0.01	<0.01
Region length total length (%)	27.8 ± 1.4	27.1 ± 1.2	0.67

^a^ Denotes significance (*p* < 0.05).

**Figure 3:**
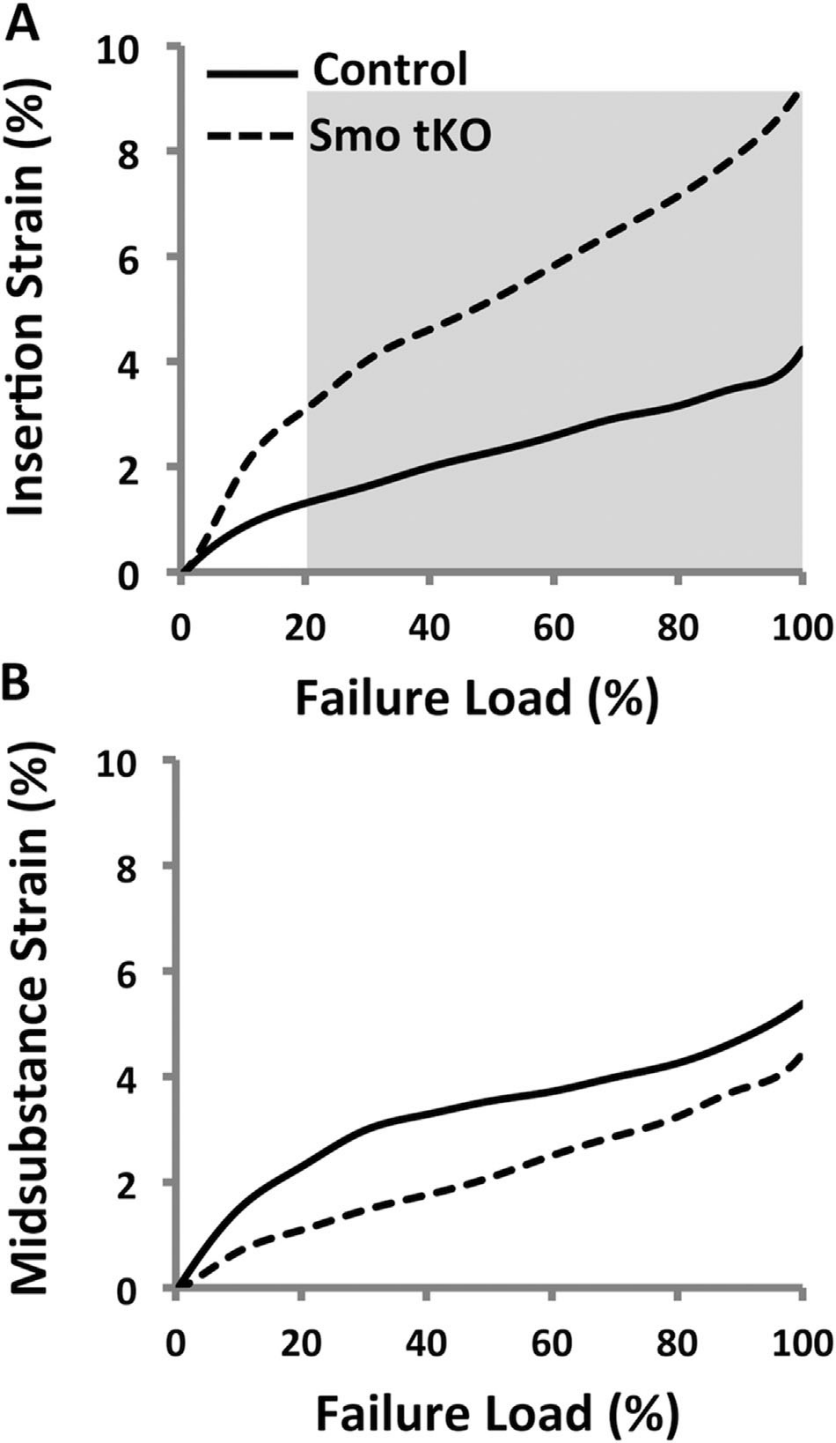
Smo tKO tendons show sub‐failure mechanical abnormalities at the distal enthesis. This figure shows the results of the B‐spline modeling of regional strain data. Analysis of β coefficients revealed that insertion strains of Smo tKO tendons were ∼2.3 fold greater than normal beyond 20% of failure load (shaded in gray, A). However, midsubstance strains did not significantly differ between groups at any load level (B).

Regional strain measurements revealed that these mechanical abnormalities were localized to the distal patellar tendon enthesis rather than the midsubstance (Fig. 3). One control sample was removed from regional strain analysis, as abnormal optical marker placement (insertion:midsubstance length ratio was more than double that of any other sample) caused it to be a statistical outlier. Tendon insertion strain was significantly increased in the Smo tKO mice. Smo tKO insertions exhibited 100% increase in failure strain (0.10 ± 0.01 mm/mm v. 0.05 ± 0.01 mm/mm, *p* < 0.01), and B‐spline modeling showed up to 123% increased strain occurring at forces as low as 20% of failure load (Fig. 3A). Coefficients β_3_–β_13_ of Smo tKO insertions were all significantly different than control coefficients (*p* < 0.05), illustrating that strain behavior was significantly increased beyond ∼20% load level (Fig. 3A). Smo tKO and control tendons showed similar midsubstance failure strain (0.05 ± 0.01 mm/mm v. 0.06 ± 0.01 mm/mm, *p* = 0.27; Table 1). B‐spline modeling also revealed that Smo tKO and control midsubstance regions experienced similar tissue strains across load levels, as no β coefficients were significantly different from one another (*p *> 0.10; Fig. 3B).

### Matrix Organization

**Figure 4:**
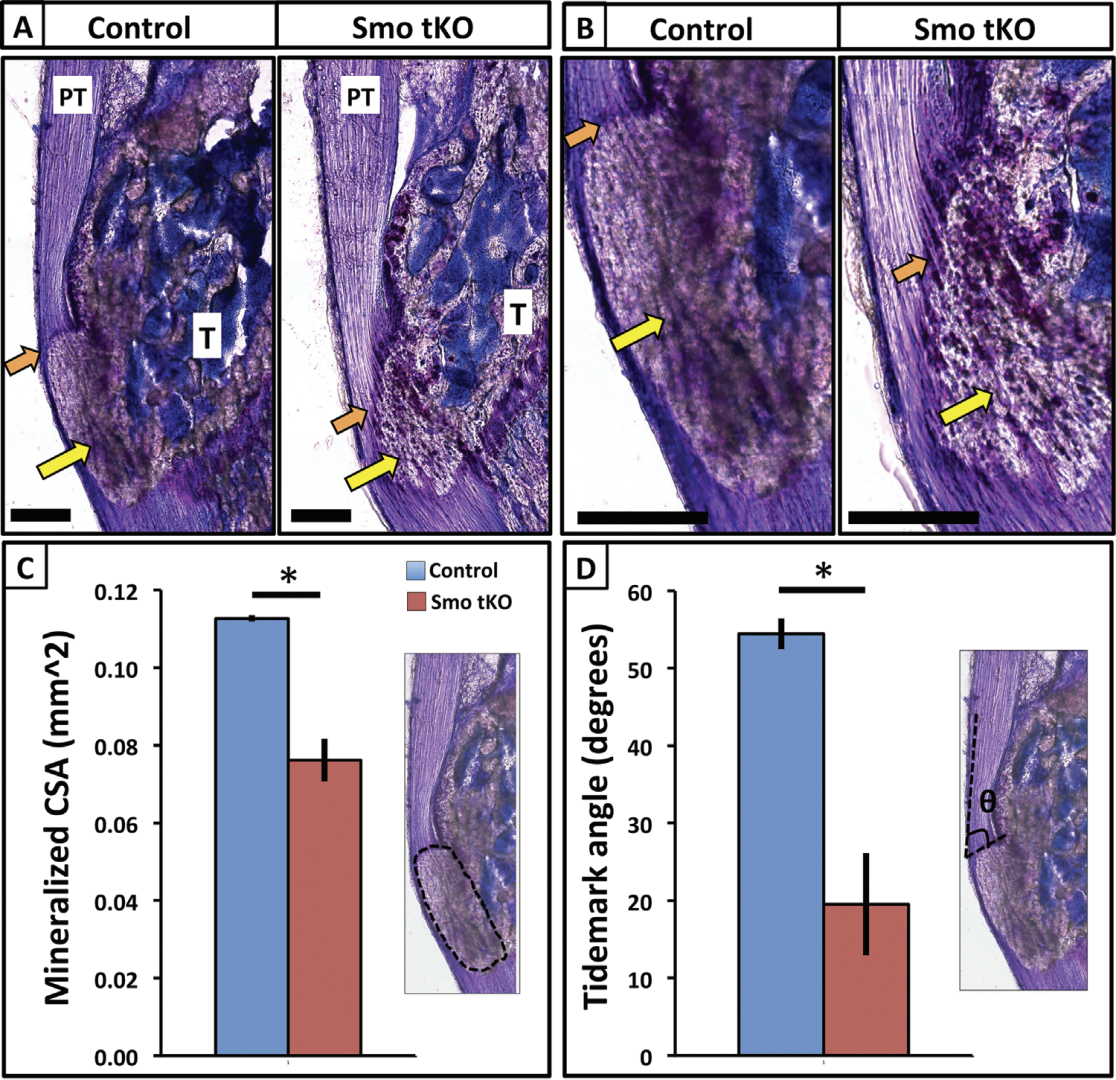
Smo tKO mice exhibit abnormalities in mineralized enthesis formation. Toluidine blue O staining of lower (A) and higher magnification (B) histology shows the different regions of the patellar tendon (PT) enthesis, including the tendon midsubstance (light purple stain), the unmineralized fibrocartilage (dark purple stain, orange arrow) and the mineralized fibrocartilage (gray hue, yellow arrow) of the tibial tuberosity on the anterior surface of the tibia (T). Although Smo tKO mice contain each of these enthesis regions, the mutants show a 32% reduction in the size of mineralized fibrocartilage (C). The smaller tibial tuberosity results in a 54% lower tidemark angle (mineralization line) with respect to the long tendon axis (D). Scale bar = 200 µm.

Ablating Hh signaling significantly disrupted the organization of the mineralized enthesis (Fig. 4). Toluidine blue O staining revealed that Smo tKO tendons contained all regions of a normal enthesis including the midsubstance, unmineralized fibrocartilage (FC), and mineralized FC (Fig. 4A and B). However, the degree of mineralization was significantly decreased, as Smo tKO tendons showed a 32% reduction in size of the mineralized FC tissue compared to controls (0.076 ± 0.006 mm^2^ v. 0.113 ± 0.001 mm^2^, *p* < 0.05; Fig. 4C). The tidemark angle was also reduced by 54% in Smo tKO tendons compared to controls (19.5 ± 6.6° v. 54.4 ± 2.0°, *p* < 0.05; Fig. 4D).

**Figure 5:**
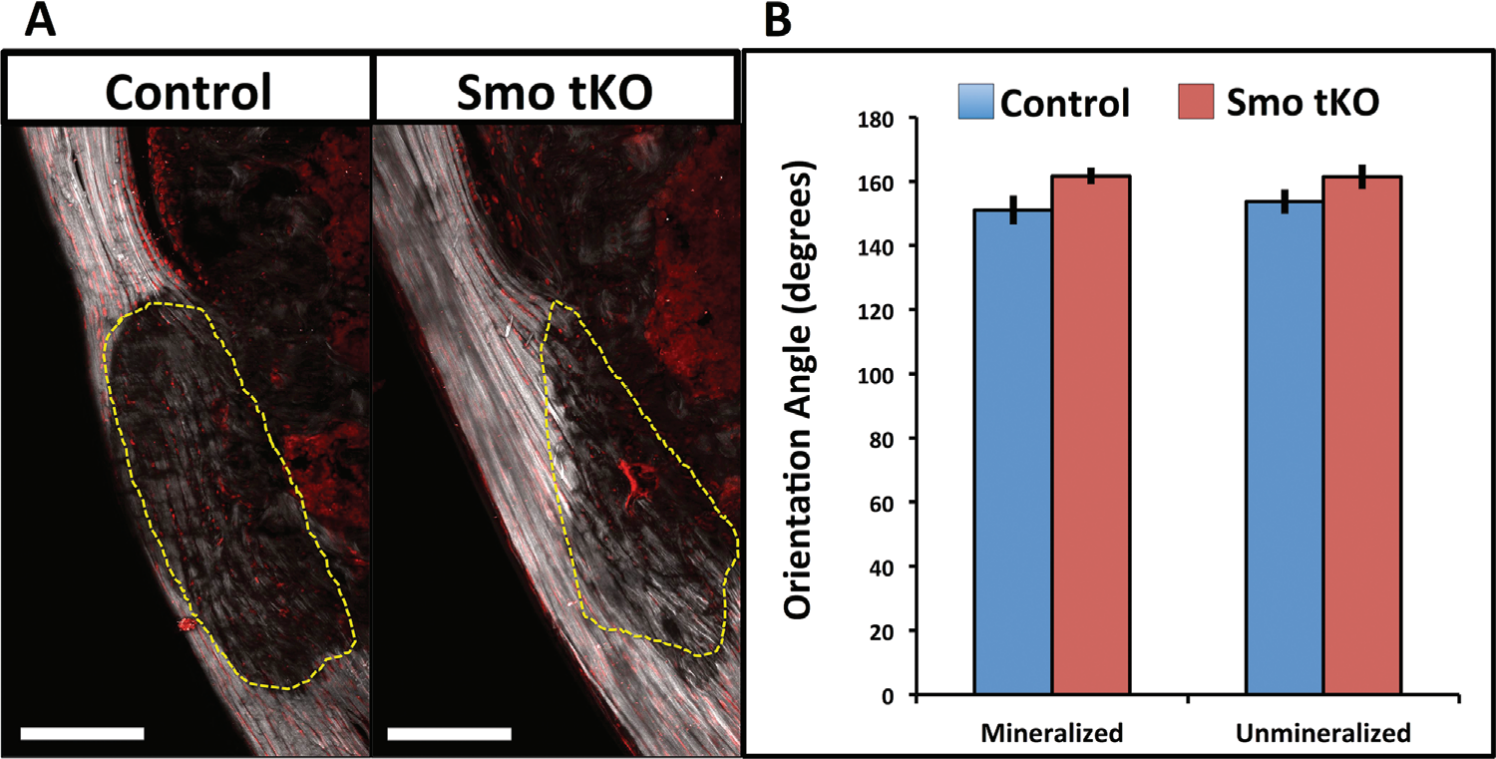
Smo tKO tendons do not exhibit changes in collagen fiber orientation. Second harmonic images (A) were used to evaluate collagen fiber alignment (with respect to the long tendon axis) in the mineralized (yellow dashed border) and unmineralized regions of the enthesis. Smo tKO tendons exhibit less collagen embedded in mineralized fibrocartilage due to the smaller size. However, this result does not affect overall fiber orientation with respect to the long tendon axis (B), as collagen fibers are similarly oriented in both unmineralized and mineralized regions of mutants and controls. Scale bar = 200 µm.

**Figure 6:**
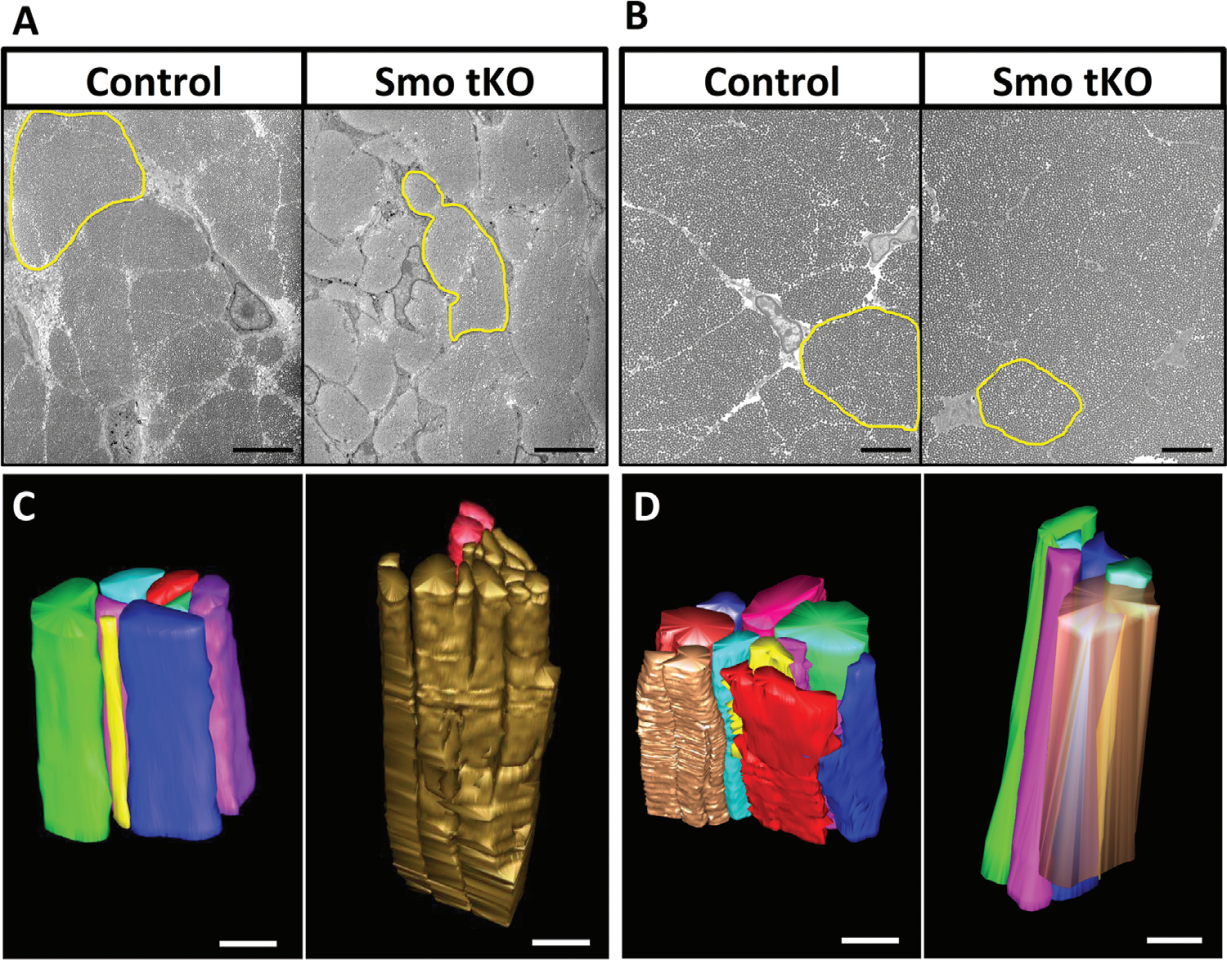
Smo tKO tendons show abnormal collagen fiber morphology. SBF‐SEM images of tendons show fibrils of the insertion (A) and midsubstance (B) are organized into fibers (boundaries between fibers are partially formed by cell membrane protrusions, seen as white in the images; sample fibers are outlined in yellow in A and B). Although fibers of control insertions are generally elliptical in shape, Smo tKO tendons exhibit abnormal polygonal morphology (A). 3D reconstructions of collagen fibers (C) further illustrate that Smo tKO insertions contain irregular fiber shape and extensive coalescence of neighboring fibers. However, fibers of the midsubstance do not appear to be different between Smo tKO and control mice (B and D), and fiber coalescence observed in the enthesis is not present in the Smo tKO midsubstance (D). Scale bars = 5 µm.

Ablating Hh signaling did not affect overall collagen fiber orientation with respect to the tendon axis (i.e., — the matrix remained parallel, to a close approximation, to the loading axis) but did alter the cross‐sectional morphology of fibers at the insertion (Fig. 5 and 6). Second harmonic generation showed that collagen fiber orientation with respect to the tendon axis did not differ between Smo tKO and control tendons in the unmineralized FC (161.7 ± 2.6° v. 151.0 ± 4.6°, *p* = 0.14) as well as the mineralized FC (161.4 ± 3.9° v. 153.6 ± 3.8°, *p* = 0.22; Fig. 5B). However, SBF‐SEM revealed that collagen fibers in the tissue proximal to the tidemark were abnormal in Smo tKO mice (Fig. 6A). While control mice exhibited distinct fibers with elliptical or kidney‐shaped cross‐sections, Smo tKO mice showed irregularly shaped fibers that coalesced more than in controls (Fig. 6A and C). Analysis of step‐through movies of SBF‐SEM images showed that collagen fibers were discontinuous and collagen fibrils within the fibers deviated from the elegant parallelism seen in wild‐type tendons (Supplementary Videos S1–S4).

## DISCUSSION

Results of this study confirm the tested hypothesis that ablating hedgehog signaling in tenocytes during development leads to abnormal biomechanics in the adult murine patellar tendon. Consistent with our previous report in 12‐week old female mice,^14^ data presented here illustrate that ablating hedgehog signaling causes 12‐week old male tendons to mature with a reduced linear stiffness and modulus (Table 1 and Fig. 2). These reductions, however, were primarily caused by increased failure displacement and strain, as tendons did not exhibit differences in ultimate failure load or stress between Smo tKO and control mice. Optically tracking surface strains of the insertion and midsubstance revealed that the increased deformation was localized to the insertion site region (Table 1 and Fig. 3). Furthermore, there were sub‐failure mechanical abnormalities as well. B‐spline modeling illustrated significant increases in strain at the insertion of Smo tKO tendons, beginning as early as 20% of failure force (Fig. 3A). While we cannot directly measure forces transmitted by the murine patellar tendon due to size limitations, patellar tendon forces measured in other animal models suggests that these tendons might experience between 21 and 40% of failure load during activities of daily living.^10^, ^28^, ^29^ The fact that Smo tKO insertions exhibited increased strain well below 40% of failure load would suggest that these tissues have functional deficits as a result of losing Hh signaling during development.

Given the increased strain at the insertion and the importance of collagen fiber alignment in maintaining tendon mechanics, we expected to see poorly organized fibers at the enthesis. Surprisingly, overall fiber orientation in the mineralized and unmineralized fibrocartilage remained largely unaffected with respect to the long tendon axis (Fig. 5A and B), indicating that collagen fiber alignment did not account for the increased strain at the enthesis. Conditional deletion of Smo within Scx‐expressing cells in the developing enthesis yielded a reduction in the amount of mineralized fibrocartilage produced by these cells, which may account for the increased strain. Since mineralized fibrocartilage will strain less than unmineralized fibrocartilage, having a lower proportion of mineralized fibrocartilage in the Smo tKO mice may yield the increased optical strain measurement. Interestingly, we identified that collagen fibers at the insertion also exhibited irregular cross‐sectional shape and coalescence of adjacent fibers (Fig. 6C). However, it is not known whether the irregular organization is a direct effect from reduced Hh signaling (indicating a novel function of Hh in fiber organization) or an indirect effect from altered mechanical loading due to the reduction of mineralized fibrocartilage in the enthesis. Future studies will investigate these potential mechanisms.

Though investigators are only beginning to elucidate the role of Hh signaling in fibrocartilage enthesis formation, they have shown that such signaling regulates chondrocyte proliferation and mineralization processes in other musculoskeletal systems. Indian hedgehog (Ihh) primarily promotes chondrocyte proliferation and mineralization of the articular cartilage.^30^, ^31^ However, Ihh‐deficient mice have significantly smaller calvaria bones and unaffected chondrocyte proliferation,^32^ illustrating that Ihh plays a more direct role in intramembranous ossification. Together, Ihh and parathyroid hormone related protein (PTHrP) regulate endochondral ossification at the growth plates of long bones through a feedback mechanism.^33^, ^34^, ^35^ Ihh serves to maintain the proliferative chondrocyte layer of the growth plate and to promote terminal differentiation into hypertrophic chondrocytes.33,^33^, ^35^ Chondrocytes then hypertrophy, apoptose and leave behind a provisional matrix to enable ingrowth of osteogenic cells and vasculature that mineralize the provided template.^36^ In this regard, Ihh‐deficient mice disrupt the Ihh‐PTHrP balance, resulting in dramatic reductions in long bone growth due to decreased chondrocyte proliferation and premature hypertrophy.^33^ PTHrP, which inhibits mineralization in articular cartilage and bone, plays an analogous role in fibrous enthesis maturation. PTHrP is highly expressed at fibrous rather than fibrocartilaginous entheses,^37^ and down‐regulation of PTHrP enhances mineralization in fibrous entheses.^38^ While we have not investigated these potential mechanisms in the mineralization of the patellar tendon enthesis, some evidence has shown that osteogenic cells infiltrate hypertrophic chondrocytes in the developing Achilles' tendon enthesis.^39^ Therefore, Ihh in fibrocartilaginous entheses may in fact function similarly to the growth plate. However, future research needs to investigate these potential mechanisms for Ihh in fibrochondrocyte proliferation, differentiation, and mineralization.

This study is not without limitations. (1) Regional strains were only measured on the tendon surface. Strain could vary substantially in the anterior‐posterior direction given that the posterior fibers of the tendon are shorter than the anterior fibers and that tendons typically fail near this posterior side of the distal insertion. However, the reduced mineralization in Smo tKO mice was more apparent in the anterior half of the tendon (Fig. 4B), suggesting this method adequately captured the biomechanical differences. (2) We did not measure proximal insertion strains at the patella. Although there was a similar phenotype of reduced collagen fiber integration and smaller patella size in the Smo tKO (Fig. S2), deviations in the strain patterns of the proximal insertion may have also contributed to the differences in total tendon mechanics. However, given that the patella and portions of the proximal region are mounted deep within an opaque grip, proximal insertion strains can only be inferred by subtracting midsubstance and insertion strains from total tendon strain. While the accuracy of this method depends on rigid fixation of the patella in the grip, our data suggest that Smo tKO mice did show approximately 72% increased patellar insertion strain compared to controls (0.29 ± 0.05 mm/mm v. 0.17 ± 0.03 mm/mm, *p* < 0.05).

Understanding how Hh signaling regulates fibrocartilage enthesis formation could be critical to improving tendon repair strategies. Surgeons often repair tendon and ligament avulsions by creating a bone tunnel through which the tissue can be passed and attached to bone. While studies have shown that this fixation improves collagen fiber integration with the bone, a lack of mineralization around the organized fibers may inhibit long‐term stability.^11^ Interestingly, our results suggest that ablating Hh signaling did not greatly affect the overall collagenous template. That is, collagen fibers in the enthesis were similarly oriented with respect to the long tendon axis. This suggests that enhancing Hh signaling at the enthesis where an aligned collagenous tissue (e.g., autograft or tissue‐engineered construct) is surgically attached might improve mineralization of the repair. However, we need a clearer understanding of how Hh signaling regulates the mineralization process compared to other musculoskeletal tissues. If Hh signaling does play a more direct role in promoting hypertrophy and subsequent mineralization, then enhancing Hh signaling at the enthesis could improve mineralization (if adult cells are competent to the Ihh ligand). However, if Hh signaling also plays a significant role in cellular proliferation, then care must be taken to evaluate how it might affect the maturation of cell‐based repairs. Given the exquisite spatiotemporal precision that leads to the formation of a normal functional enthesis, investigators need to continue to examine the mechanisms of Hh signaling in fibrocartilage maturation. By understanding these processes and other mechanisms in tendon enthesis formation, we can begin to identify what environment (matrix content, cell phenotype, mechanical loading) is required for activated Hh signaling to be effective, thus improving the likelihood of developing a successful repair. By identifying and prioritizing these necessary factors, we can begin to establish biological design criteria that achieve mechanical design criteria and that lead to more function tissue repairs.^40^

## AUTHORS’ CONTRIBUTIONS

Research Design: APB, NAD, CFL, CW, MR, RJ, and DLB

Data Acquisition: APB, LAS, YL, CFL, HL

Data Analysis/Interpretation: APB, NAD, MB, JTS, KEK, DLB

## Supporting information

Additional supporting information may be found in the online version of this article at the publisher's web‐site.

**Supporting Information Figure S1**: Basis Spline Coefficient Intervals. Mechanical data were modeled using basis spline modeling with cubic polynomials and 9 interior knots.

**Supporting Information Figure S2**: Hh Ablation May Also Reduce Mineralization in the Patella. Smo tKO mice show a similar phenotype at the patella as seen in the tibial insertion.

**Supporting Information Video S1**: Step through movies of SBF‐SEM.

**Supporting Information Video S2**.

**Supporting Information Video S3**.

**Supporting Information Video S4**.
