# Supplementary figures and images for: Ablating hedgehog signaling in tenocytes during development impairs biomechanics and matrix organization of the adult murine patellar tendon enthesis
Source: J Orthop Res. 2015 Apr 14;33(8):1142–51. doi: 10.1002/jor.22899 (PMC4706742; doi:10.1002/jor.22899)

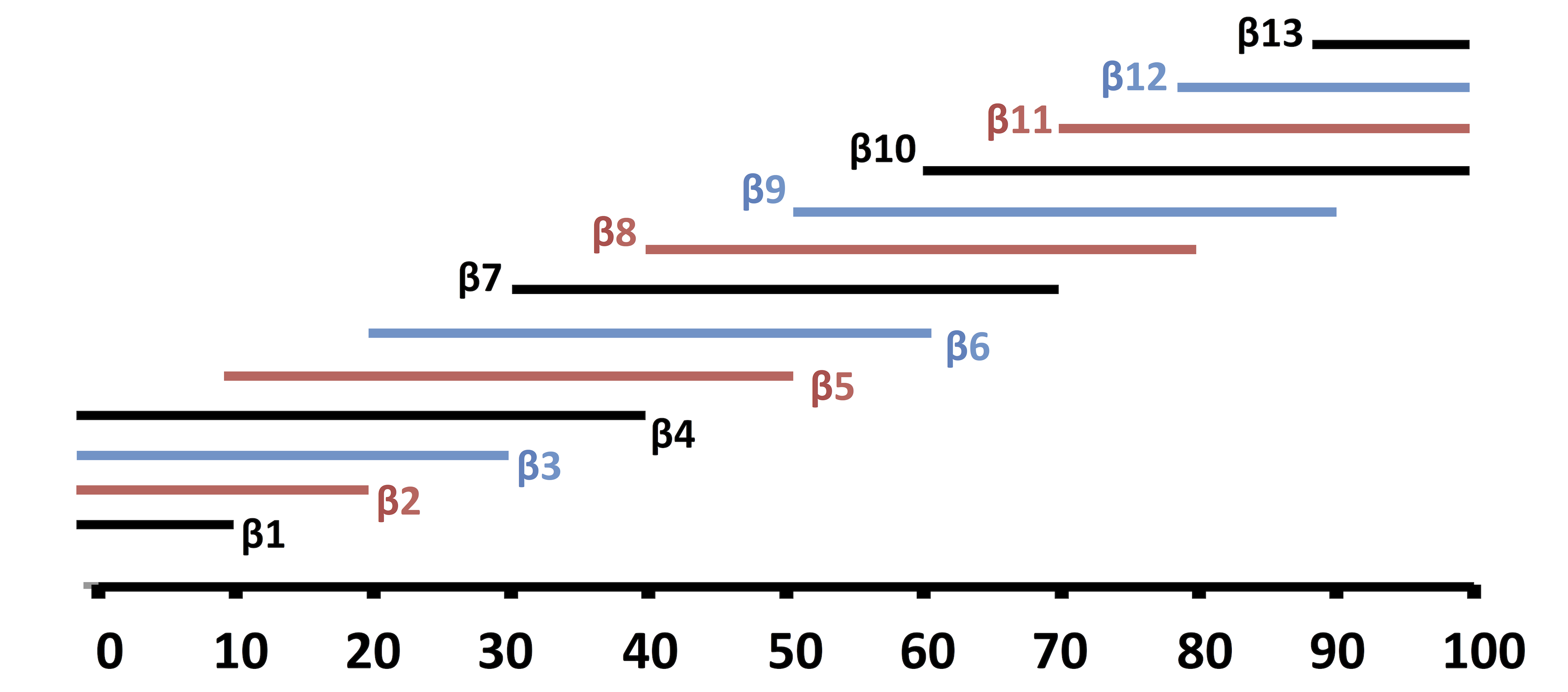

Supplement: Supplementary file 1 — Supporting Information Figure S1: Basis Spline Coefficient Intervals. Mechanical data were modeled using basis spline modeling with cubic polynomials and 9 interior knots. [file JOR-33-1142-s001.tif]

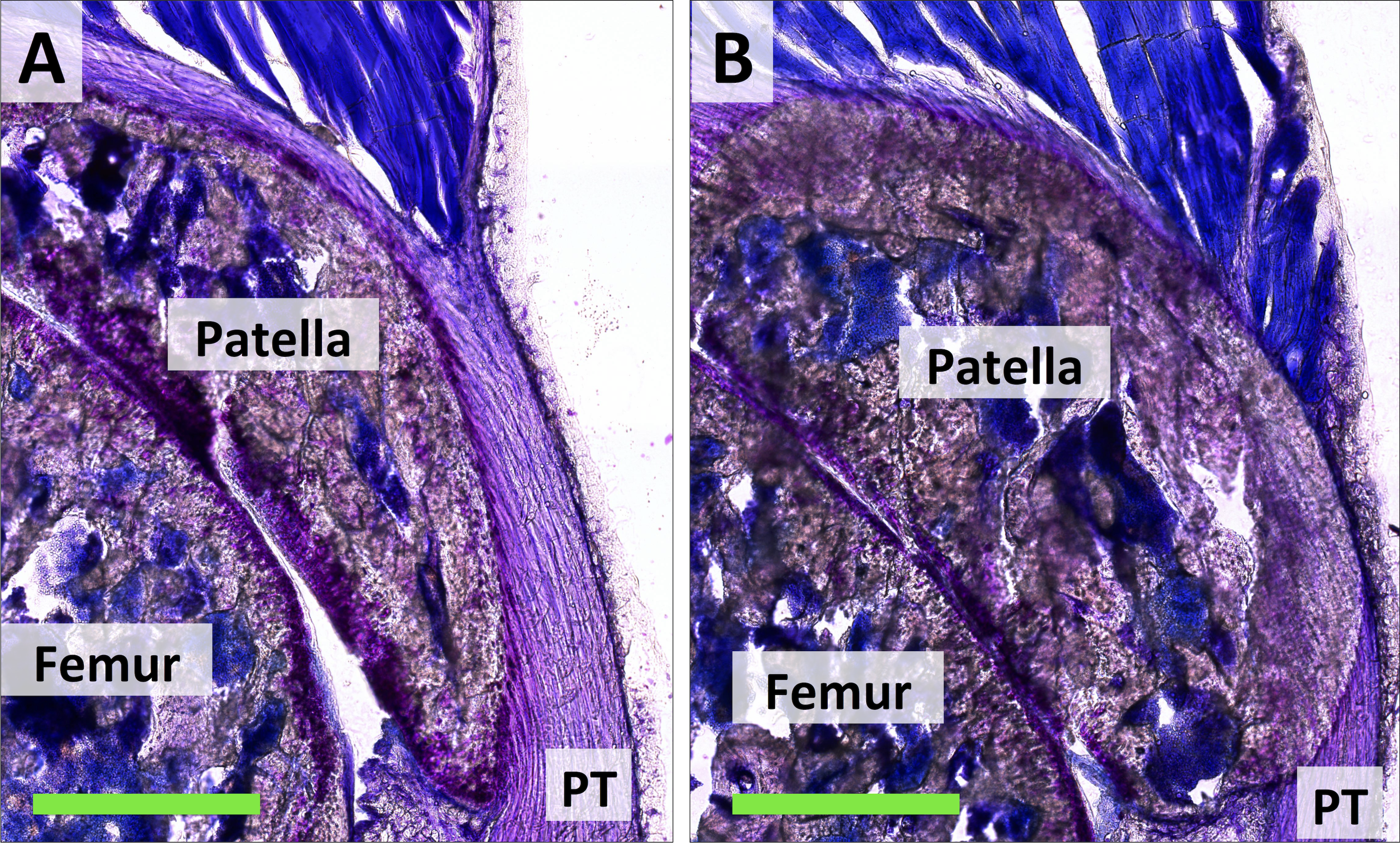

Supplement: Supplementary file 2 — Supporting Information Figure S2: Hh Ablation May Also Reduce Mineralization in the Patella. Smo tKO mice show a similar phenotype at the patella as seen in the tibial insertion. [file JOR-33-1142-s002.tif]
